# Biofilm formation is a risk factor for mortality in patients with *Candida albicans* bloodstream infection—Scotland, 2012–2013

**DOI:** 10.1016/j.cmi.2015.09.018

**Published:** 2016-01

**Authors:** R. Rajendran, L. Sherry, C.J. Nile, A. Sherriff, E.M. Johnson, M.F. Hanson, C. Williams, C.A. Munro, B.J. Jones, G. Ramage

**Affiliations:** 1) School of Medicine, College of Medical, Veterinary and Life Sciences, University of Glasgow, Glasgow, UK; 2) Public Health England, Southwest Laboratory, Bristol, UK; 3) NHS Lothian, Edinburgh, UK; 4) University of the West of Scotland, Glasgow, UK; 5) University of Aberdeen, Aberdeen, UK; 6) Microbiology Department, Glasgow Royal Infirmary, Glasgow, UK

**Keywords:** Antifungal, biofilm, *Candida albicans*, *Candida glabrata*, candidaemia, catheters, drug resistance

## Abstract

Bloodstream infections caused by *Candida* species remain a significant cause of morbidity and mortality in hospitalized patients. Biofilm formation by *Candida* species is an important virulence factor for disease pathogenesis. A prospective analysis of patients with *Candida* bloodstream infection (*n* = 217) in Scotland (2012–2013) was performed to assess the risk factors associated with patient mortality, in particular the impact of biofilm formation. *Candida* bloodstream isolates (*n* = 280) and clinical records for 157 patients were collected through 11 different health boards across Scotland. Biofilm formation by clinical isolates was assessed *in vitro* with standard biomass assays. The role of biofilm phenotype on treatment efficacy was also evaluated *in vitro* by treating preformed biofilms with fixed concentrations of different classes of antifungal. Available mortality data for 134 patients showed that the 30-day candidaemia case mortality rate was 41%, with predisposing factors including patient age and catheter removal. Multivariate Cox regression survival analysis for 42 patients showed a significantly higher mortality rate for *Candida albicans* infection than for *Candida glabrata* infection. Biofilm-forming ability was significantly associated with *C. albicans* mortality (34 patients). Finally, *in vitro* antifungal sensitivity testing showed that low biofilm formers and high biofilm formers were differentially affected by azoles and echinocandins, but not by polyenes. This study provides further evidence that the biofilm phenotype represents a significant clinical entity, and that isolates with this phenotype differentially respond to antifungal therapy *in vitro*. Collectively, these findings show that greater clinical understanding is required with respect to *Candida* biofilm infections, and the implications of isolate heterogeneity.

## Introduction

*Candida* species bloodstream infection (BSI) remains a significant cause of morbidity and mortality [1], [2]. In the USA, *Candida* species are ranked as the fourth most common organisms responsible for all BSIs, and the third most common within the intensive-care unit [2], a clinical environment that is highly dependent on intravascular lines. *Candida* BSI is often associated with the ability of *Candida* to form biofilms on indwelling medical devices, such as central venous catheters (CVCs) and prostheses [3], [4]. *Candida albicans* remains one of the most important candidal pathogens in this context, owing in part to its greater capacity to form biofilms [5], and this has profound consequences for the clinical outcome of BSI. Therefore, removal of catheters is advocated to improve survival rates, on the basis of meta-analysis evidence from current guidelines [6], [7].

Retrospective studies have used multivariate approaches to attempt to analyse the risk factors associated with patients with *Candida* BSI. Biofilm formation has been reported as an independent predictor of mortality, in addition to inadequate antifungal therapy and APACHE III scores [8]. Analysis of the association of mortality with biofilm-forming ability demonstrated that both *C. albicans* and *Candida parapsilosis* were associated with increased mortality. A subsequent prospective case–control study showed that *Candida* BSI biofilm-forming isolates could be independently predicted by the presence of CVCs, urinary catheters, total parenteral nutrition, and diabetes mellitus [9]. Moreover, the hospital length of stay and cost of antifungal therapy were also greater in those with biofilm-forming isolates, and these patients had a greater risk of hospital mortality (OR 1.77). However, these studies used binary categorization of biofilm formation, i.e. biofilm formers or non-formers, on the basis of *in vitro* bioassays. Our group has recently reported that biofilm formation by *C. albicans* is heterogeneous, and that, rather than biofilm formation being a binary function, it can be considered on a spectrum or within defined categories [10]. Therefore, there remains a gap in our knowledge as to whether patients with isolates defined as low biofilm formers (LBFs) or high biofilm formers (HBFs) within the spectrum have differential clinical outcomes. The aim of this study was therefore to investigate the impact of biofilm formation by *Candida* species on the clinical outcomes of BSI in a defined Scottish cohort.

## Patients and methods

### Patients and variables

A retrospective study of all cases of *Candida* BSI was carried out within Scotland under NHS Caldicott Guardian approval from March 2012 to February 2013. *Candida* BSI was reported in 217 patients from 11 different health boards; clinical data were obtained from 157 patients. The complete datasets of patient demographics, underlying medical conditions and details of antimicrobial therapy were collected through a review of the medical case notes in each health board. Where available (134 patients), patient outcomes were followed from the first positive blood culture until 30 days or death, and clinical details, including the presence of indwelling medical devices in the 30 days prior to the detection of *Candida* BSI were also collected. All data were collected and stored electronically within a database (Excel, Microsoft).

### Isolate collection

Blood cultures from 217 patients were processed according to routine standard operating procedures in each of the referring laboratories. When available, multiple isolates were collected from some of these patients within the observation period of 30 days. All clinical isolates obtained during this period were independently identified by the use of Colorex *Candida* chromogenic plates (E&O Laboratories, Bonnybridge, UK), as confirmed by matrix-assisted laser desorption ionization time-of-flight mass spectrometry at the Public Health England Southwest Laboratory (Bristol), and were stored in Microbank vials (Pro-Lab Diagnostics, Bromborough, UK) at −80°C until further use. These isolates were subcultured on Sabouraud's dextrose agar (Sigma-Aldrich, Poole, UK). Plates were incubated at 30°C for 48 h, and maintained at 4°C.

### Biofilm formation

*Candida* species biofilms were grown according to our established protocols for 24 h [11], and the biomass of each isolate was assessed with the crystal violet (CV), XTT and SYTO9 assays, as previously reported [10], [12], [13]; isolates were grouped on the basis of their level of biomass distribution (optical density (OD)_570_ _nm_ values). Isolates within the first quartile (Q1) were classed as LBFs, isolates with a biomass greater than the third quartile (Q3) were classed as HBFs, and those in between were classed as intermediate biofilm formers (IBFs) (second quartile (Q2)) [10]. The susceptibilities of *C. albicans* biofilm formation to different classes of antifungal were also assessed, with 24-h biofilms being treated with either 2 mg/L or 200 mg/L voriconazole, caspofungin or amphotericin B for 24 h. Following treatment, the proportional viability was compared with that untreated control by use of an XTT metabolic assay [13].

### Statistical analysis

Initially, all data were numerically coded and labelled for each variable, and analysed with SPSS software (SPSS, Chicago, IL, USA). Categorical variables were compared between groups by use of the two-tailed *χ*^2^ test or Fisher’s exact test, as appropriate. Two groups of any continuous variables were compared by the use of Student’s *t*-test or the Mann–Whitney *U*-test, as appropriate. Pairwise correlations between biofilm assays were determined by calculating two-tailed Pearson correlation coefficients. The survival distribution function was estimated with the Kaplan–Meier method, and a non-parametric log-rank test was used to compare the survival curves among the different groups. Variables showing a significant association with survival according to Student’s *t*-test or the *χ*^2^ test were included in subsequent univariate and multivariate Cox regression analyses, to generate the survival curves, hazard ratios (HRs), and 95% CIs.

## Results

### Incidence and mortality associated with *Candida* species

Data from the most recent (2011) census (http://www.scrol.gov.uk/scrol/common/home.jsp) list the population of Scotland as 5 295 403. The population-based incidence of BSI in Scotland can therefore be calculated as 4.1 per 100 000 population. Of the 280 isolates collected in this study from 217 patients, 115 were found to be *C. albicans* and 98 were *Candida glabrata*. Of the 134 cases for which patient mortality data were available, the overall crude mortality rate was 41%, which was primarily associated with *C. albicans* (47.3%), followed by *C. glabrata* (34.5%) and other species (18.2%).

### Clinical parameters influencing patient mortality

Initially, we assessed the influence of different clinical variables and underlying conditions on patient mortality (Table 1). The results showed that patient age was significantly associated with mortality (p 0.023). Other variables, including underlying clinical conditions, i.e. diabetes, liver disease, autoimmune disorders, and others, were also assessed, and found not to be statistically associated with patient mortality. Moreover, 96.4% of patients had lines *in situ*, including 93% of patients with CVCs. Further analysis showed an association between line removal after diagnosis of *Candida* BSI and mortality (p 0.032).

### Relationship between biofilm formation and mortality

**Fig. 1:**
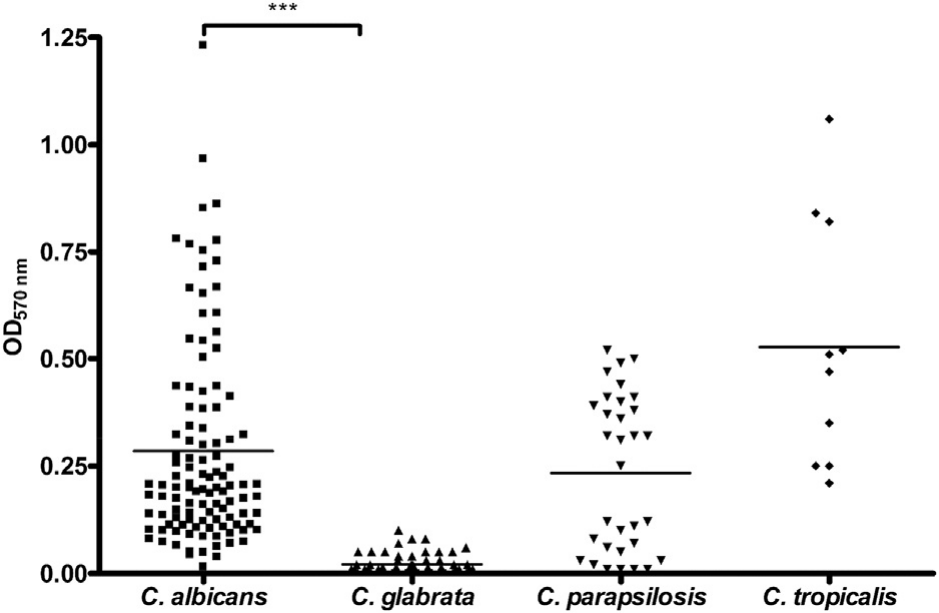
Biofilm formation by *Candida* species. *Candida* bloodstream isolates were evaluated for biofilm formation with standardized methods. *Candida* isolates standardized (1 × 10^6^ cells/mL) in RPMI-1640 were grown in flat-bottomed 96-well microtitre plates for 24 h at 37°C. Mature biofilms were carefully washed with phosphate-buffered saline and allowed to air dry, and biomass quantified by staining with 0.05% w/v crystal violet solution. The biofilms were washed and destained with 100% ethanol. Biomass was quantified spectrophotometrically by reading the optical density (OD) at 570 nm in a microtitre plate reader (FluoStar Omega BMG Labtech, Aylesbury, UK). Six replicates were used for each isolate, and the mean of each is represented. ***p <0.0001.

Biofilm formation (biomass) by different *Candida* species was assessed with CV, XTT and SYTO9 assays of *C. albicans* (*n* = 107), *C. glabrata* (*n* = 96), *C. parapsilosis* (*n* = 32), and *Candida tropicalis* (*n* = 10). When the different biomass assays were compared, significant positive correlations were found for *C. albicans* between CV and XTT (*r* = 0.8), between CV and SYTO9 (*r* = 0.6), and between XTT and SYTO9 (*r* = 0.4) (Fig. S1). On the basis of this, CV was used for further categorization of the isolates. Fig. 1 shows that biofilm formation by different *Candida* species was heterogeneous, irrespective of the species tested. Isolates were categorized as LBFs or HBFs if their CV absorbance was less than Q1 (OD_570__nm_ = 0.15) or greater than Q3 (OD_570__nm_ = 0.3), respectively. Those isolates between Q1 and Q3 were defined as IBFs. *C. albicans*, *C. parapsilosis* and *C. tropicalis* were found to be HBFs (*n* = 35, *n* = 16, and *n* = 5, respectively), IBFs (*n* = 36, *n* = 3, and *n* = 5, respectively), or LBFs (*n* = 35 *n* = 13, and *n* = 0, respectively), whereas *C. glabrata* organisms were found to be only LBFs (*n* = 98).

**Fig. 2:**
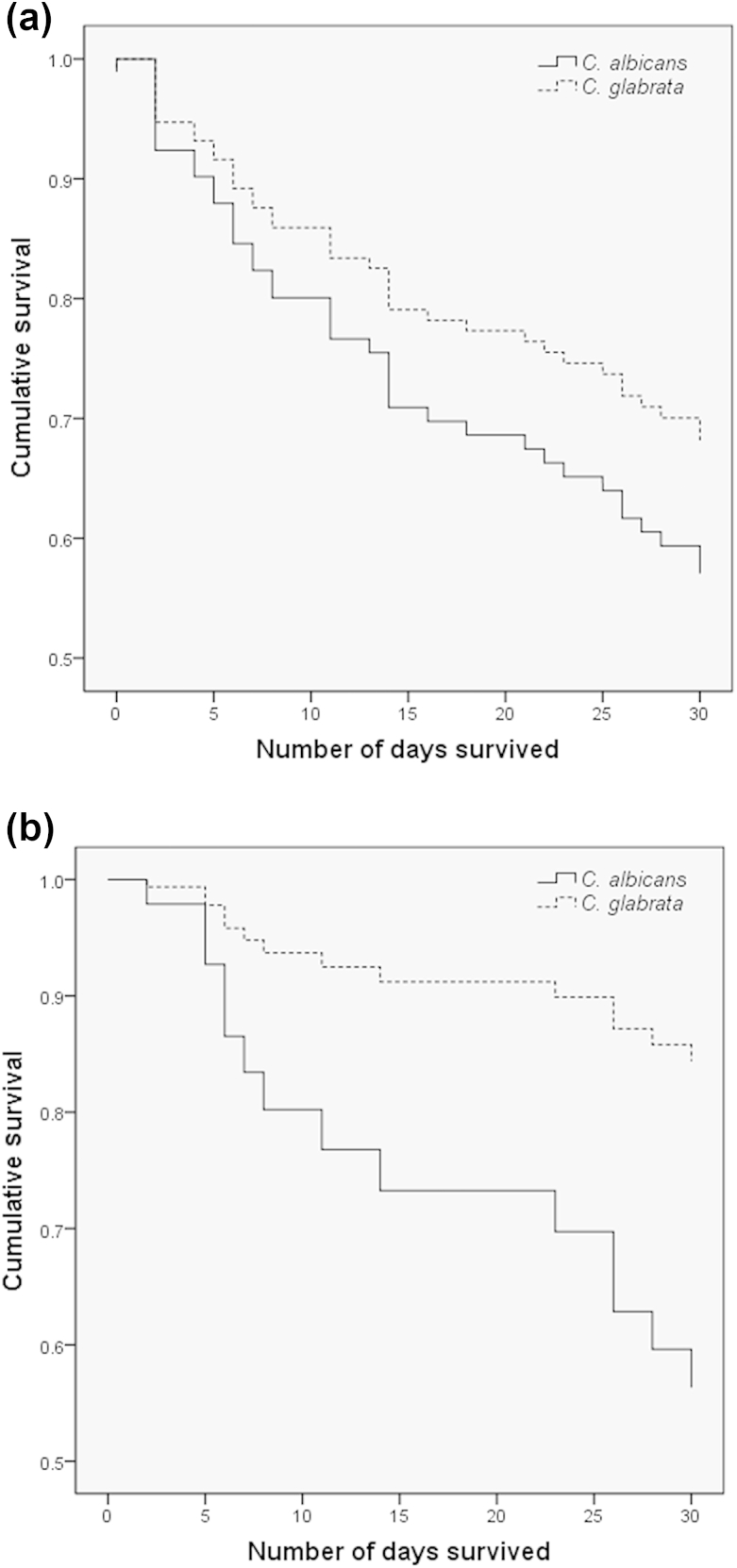
Survival of patients with *Candida albicans* and *Candida glabrata* infection. Survival of patients infected with *C. albicans* (solid line) and *C. glabrata* (dotted line) was monitored over a period of 30 days from the first *Candida*-positive blood culture. Cox regression plots, adjusted only for patient age (*n* = 95) (a) or for age and catheter removal (*n* = 42) (b), in patients with *C. albicans* and *C. glabrata* infection are shown. Comparison between these curves showed a statistically significant difference in the mortality rate in (b) (p <0.05).

**Fig. 3:**
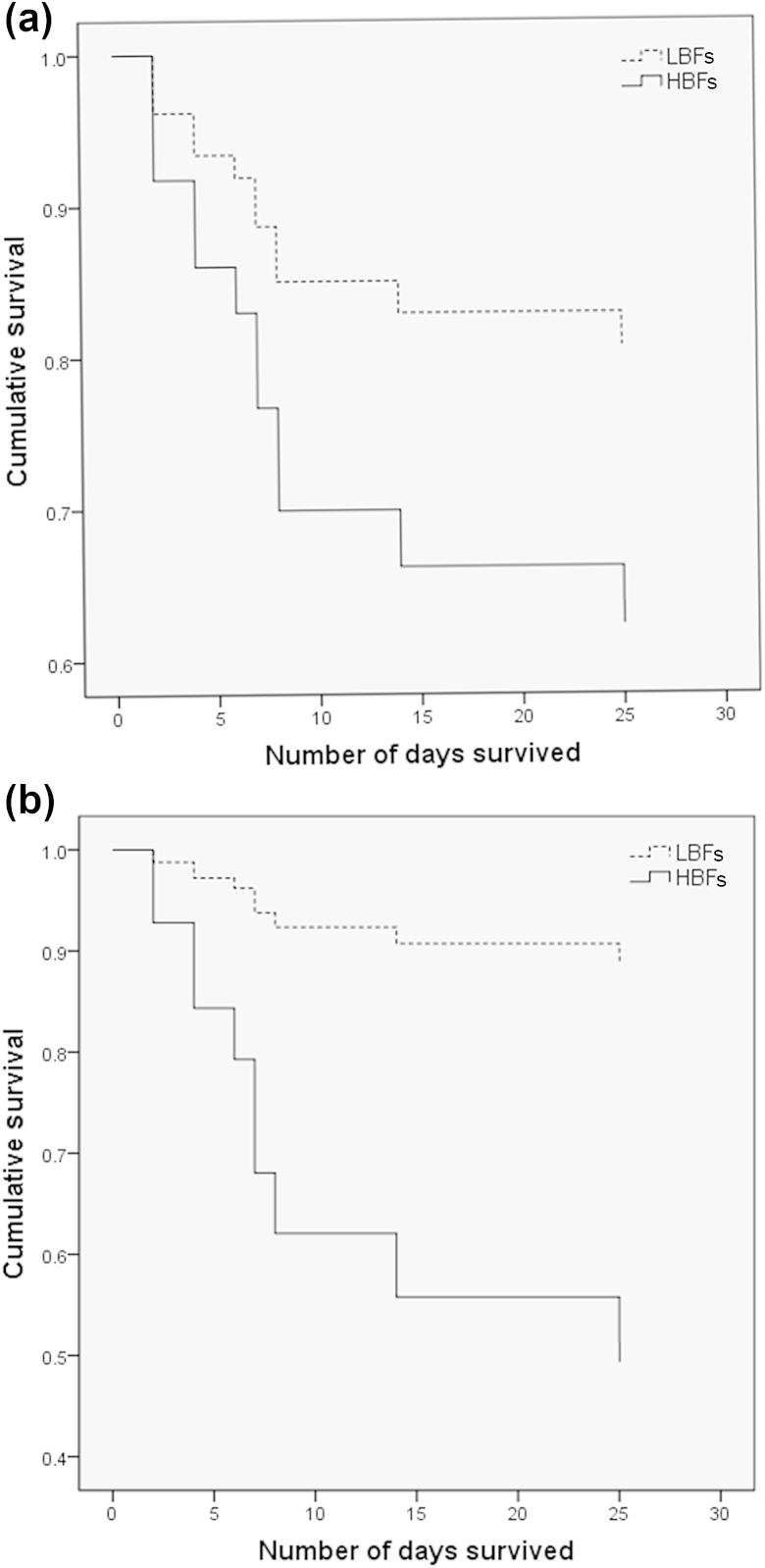
Survival of patients with *Candida albicans* high biofilm formers (HBFs) and low biofilm formers (LBFs). Survival of patients infected with *C. albicans* HBFs (*n* = 17) and LBFs (*n* = 17) was monitored over a period of 30 days from the first *Candida*-positive blood culture. Cox regression plots adjusted for (a) age only (*n* = 34) or (b) age and parenteral nutrition (*n* = 28) in patients with *C. albicans* HBFs (solid line) and LBFs (dotted line) are shown. Comparison between these curves showed a statistically significant difference in the mortality rate in (b) (p <0.05).

Given the significant disparity in biofilm formation between *C. albicans* and *C. glabrata* (p <0.0001), we analysed patient mortality (*n* = 95) with respect to these two groups on the basis of 30-day survival from the first positive blood culture (Fig. 2). Comparison of these by the use of Cox regression analysis adjusted for age showed a trend for higher mortality with *C. albicans* than with *C. glabrata* (p 0.260) (Fig. 2a), and, after adjustment for catheter line removal from patients (*n* = 42), showed a significant difference between these curves (Fig. 2b; p 0.048, HR 3.4, 95% CI 0.99–11.47). Next, we investigated whether the levels of biofilm formation by *C. albicans* showed an association with mortality, by specifically evaluating isolates defined as LBFs (*n* = 17) and HBFs (*n* = 17). The Cox regression plots adjusted for age showed a trend for a higher mortality rate with HBFs than with LBFs (p 0.192) (Fig. 3a). A previous study has shown that administration of parenteral nutrition induces *C. albicans* germination and biofilm formation [14]. Therefore, we performed analysis with adjustments for administration of parenteral nutrition, and these revealed a significant difference in survival between the LBF and HBF groups (Fig. 3b; p 0.024, HR 5.99, 95% CI 1.3–28.3).

### Biofilm sensitivity to antifungals

**Fig. 4:**
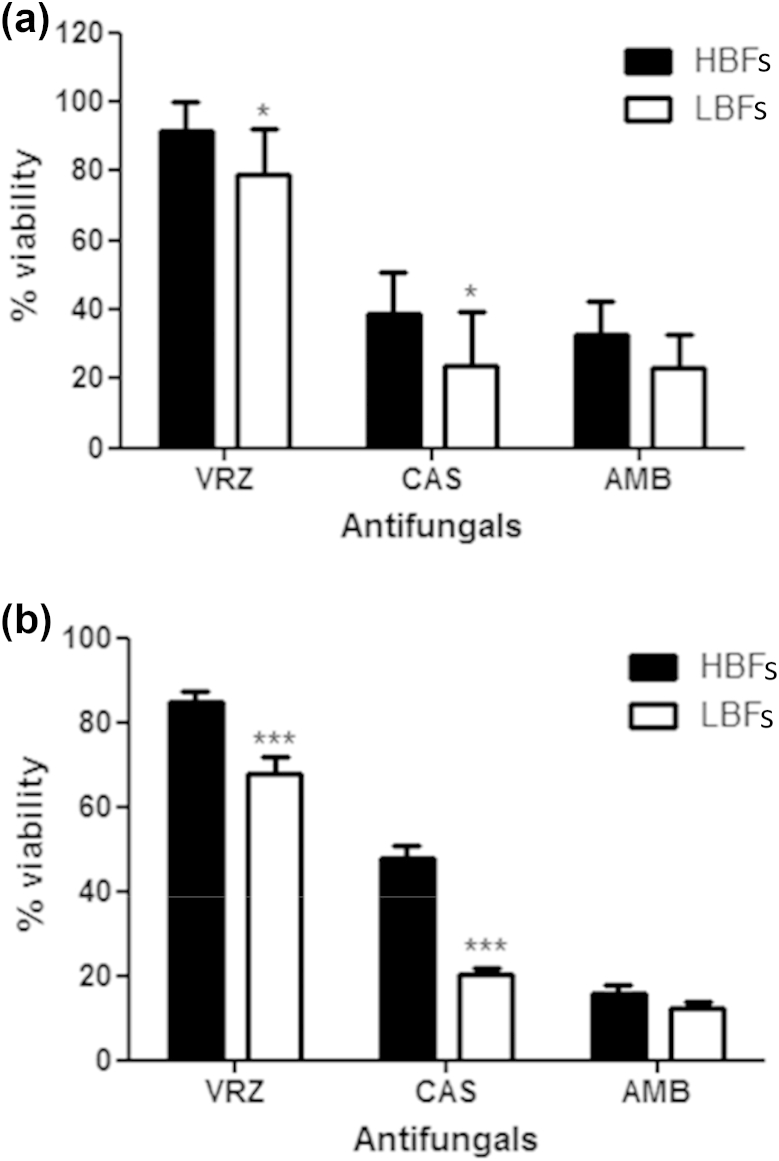
Impact of *Candida albicans* biofilm formation on antifungal susceptibility. Ten low biofilm formers (LBFs) and high biofilm formers (HBFs) were standardized to 1 × 10^6^ cells/mL in RPMI-1640, and grown as biofilms in flat-bottomed 96-well microtitre plates for 24 h. Biofilms were washed with phosphate-buffered saline before being treated with 2 mg/L (a) or 200 mg/L (b) voriconazole (VRZ), caspofungin (CAS), and amphotericin B (AMB). After incubation for 24 h, metabolic activity was measured with the XTT assay, with optical density being read at 492 nm. Percentage viability was calculated relative to untreated controls, and data are presented as mean ± standard deviation. Eight replicates were used for each isolate, and repeated on two separate occasions. *p <0.05, ***p <0.001.

On the basis of these data, and given the positive correlations between biofilm formation and mortality, we tested *C. albicans* LBFs (*n* = 10) and HBFs (*n* = 10) for their response to azoles (voriconazole), polyenes (amphotericin B) and echinocandins (caspofungin) at low (2 mg/L) and high (200 mg/L) concentrations. Although both 2 mg/L and 200 mg/L voriconazole were equally ineffective against mature HBF and LBF biofilms (Fig. 4a,b), a significant difference in overall activity was observed between HBFs and LBFs at both 2 mg/L (p <0.05) and 200 mg/L (p <0.001). Conversely, caspofungin and amphotericin B were effective against both HBFs and LBFs, although the levels of biofilm formation significantly impacted on caspofungin sensitivity (2 mg/L, p <0.05; 200 mg/L, p <0.001). Furthermore, a paradoxical effect was found for caspofungin with HBFs, whereby 200 mg/L was significantly less effective than 2 mg/L (p <0.05). Conversely, no such effect was found in LBFs. Amphotericin B was shown to be equally effective against LBFs and HBFs, although a significant difference was found between 2 mg/L and 200 mg/L (HBFs, p <0.0005; LBFs, p <0.005).

## Discussion

A retrospective analysis of patients with *Candida* BSI in Scotland was performed to determine the risk factors associated with mortality in the defined patient cohort. We report an adverse influence of biofilm formation by *Candida* species on the clinical outcomes of patients with *C. albicans* BSI.

The incidence of *Candida* BSI from this study in 2012–2013 (4.1 cases per 100 000 population per year) is comparable to that in 2005–2006 (4.8 cases per 100 000 population per year) [15]. The difference could be explained by an increasing population and by inaccurate reporting of the cases, and is therefore likely to represent a minimum estimate of the incidence of *Candida* BSI in Scotland. Our previous study reported that *C. albicans* was most prevalent within the population (50%), followed by *C. glabrata* (21%) [15]. The data from this study demonstrate a changing epidemiology, with a notable decrease in the incidence of *C. albicans* (41%) and a concurrent increase in the incidence of *C. glabrata* (35%). The reason for this is uncertain, but the overuse of azoles may have inadvertently selected *C. glabrata*, which has reduced sensitivity to fluconazole. The use of matrix-assisted laser desorption ionization time-of-flight mass spectrometry in addition to standard laboratory identification may have improved the isolation rate of this organism [16], [17], [18].

Among the patients with a BSI, we found a mortality rate of 41%, for which a number of risk factors were identified. Risk factors such as neutropenia, glucocorticosteroids, parenteral nutrition and CVC use were reported to be associated with *Candida* BSI [19]. In addition to *Candida* infection, the patient age covariate had a significant influence on patient mortality. It is of significance, however, that our data show that removal of catheter lines after diagnosis of candidaemia significantly improves the clinical outcome (p 0.032). These data support the current guidelines for the management of catheter-associated infection and their clinical management; that is, where possible, the catheter should be removed in non-neutropenic patients [7], [20], [21]. In a prospective randomized trial, it was shown that the removal of a catheter within the first 24 h of candidaemia resulted in a shorter duration of candidaemia [22]. Furthermore, a recent meta-analysis reported that removal of the CVC is associated with decreased mortality [6]. Biofilms are relatively refractory to antifungal therapy [3], [11]; therefore, unsurprisingly, inadequate antifungal therapy (OR 2.35, p 0.03) and biofilm formation (OR 2.33, p 0.007) were reported to be independent predictors of mortality in candidaemia patients [8]. Biofilm formation by clinical isolates of different *Candida* species was found to be highly variable, particularly between *C. albicans* and *C. glabrata*. Survival analysis was carried out to investigate the impact of their biofilm formation on clinical outcome, with Cox regression showing a trend for *C. albicans* to be more associated with mortality (p 0.026). After adjustment for the removal of lines, a significant difference in mortality was observed (p <0.05). These data are different from those previously reported, where no significant differences in survival between patients with *C. glabrata* and with *C. albicans* BSI was observed [23]. In addition, Cox regression analysis with the *C. albicans* HBF (41.2% mortality) and LBF (35.2% mortality) groups adjusted for patient age showed a trend for a lower survival rate with HBFs than with LBFs, which is in accordance with a previous study showing 51.2% mortality in the biofilm-forming group, as compared with 31.7% in the non-biofilm-forming group (p 0.004) [9]. Parenteral nutrition, including lipid emulsion, has been shown to induce *C. albicans* germination and increase biofilm formation in indwelling catheters [14]. Therefore, Cox regression analysis with adjustment for parenteral nutrition was performed, and revealed a significant impact on biofilm-related mortality. However, a caveat to the interpretation of these data is the low sample numbers, owing to the availability of complete datasets.

Given that biofilm formation by clinical isolates is heterogeneous and has an impact on patient mortality, we tested their antifungal sensitivity to determine whether it was affected. Biofilms were treated with antifungals at a clinically relevant concentration of 2 mg/L and another concentration that is potentially useful in antifungal lock therapy (200 mg/L) [24]. Our data illustrate a significant difference between HBFs and LBFs in sensitivity to voriconazole and caspofungin. However, amphotericin B is equally effective against both groups. Whereas the clinical data show that azoles were used extensively to treat 82.2% of patients in this study, our data show that this class of antifungal is less effective against matured biofilms. When echinocandins were compared with polyenes, the removal of the catheter showed no improved time to mycological eradication, possibly because of the effectiveness of both antifungal agents against biofilms [25]. Furthermore, with HBFs, we found a paradoxical effect of lower percentage kill with a higher concentration of caspofungin (200 mg/mL) than with 2 mg/L, *in vitro* (Fig. 4b). The exact mechanism causing this paradoxical effect with HBFs and its clinical relevance are unknown.

In summary, *C. albicans* remains a predominant *Candida* species associated with high mortality in candidaemia patients, and, within this species, biofilm heterogeneity has a direct impact on patient survival, and potentially on antifungal sensitivities, based on *in vitro* studies. These findings highlight the importance of biofilm stratification in the clinical management of candidaemia cases, to determine whether they should be managed with azoles or other fungicidal classes of antifungal agent. Moreover, understanding the genetic basis of these isolates will enable us to devise and develop a biomarker for biofilm-related *Candida* BSI, and to create more appropriate antifungal therapies, which, collectively, will facilitate improved clinical outcomes.

## Transparency declaration

G. Ramage, B.J. Jones and C. Williams has received research grants and acted as a speaker for Gilead, MSD and Astellas.

## Figures and Tables

**Table 1:** Variables stratified according to the survival or death status at the 30-day endpoint for the 134 patients studied

Variables	% Survived	% Died	p
Age (years), mean ± SD	58.6 ± 20.7 (79)	66.9 ± 20.0 (55)	**0.023**
Male sex	48.1 (79)	83.6 (55)	0.096
Diabetes	25.6 (78)	32.7 (55)	0.437
Surgery	63.6 (22)	50.0 (18)	0.523
Radiotherapy	37.5 (16)	30.8 (13)	1
Chemotherapy	64.3 (14)	56.3 (16)	0.722
Solid organ transplant	5.1 (79)	0.0 (55)	0.144
Metastatic	61.1 (18)	85.7 (14)	0.235
Solid tumour	32.1 (78)	38.9 (54)	0.460
Autoimmune or genetic disorder	11.4 (79)	7.4 (54)	0.559
Renal failure	32.9 (73)	42.2 (45)	0.330
Liver disease	8.3 (72)	14.3 (49)	0.374
Alcohol abuse	10.5 (76)	15.4 (52)	0.428
ICU admission	19.7 (76)	28.8 (52)	0.289
Parenteral nutrition	43.1 (72)	37.3 (51)	0.579
Line removed	82.2 (45)	55.0 (20)	**0.032**
Antifungals in previous 3 months	27.5 (69)	14.0 (50)	0.115

Values in parentheses indicate the total no. of patients assessed for each variable. Bold type indicates a significant result.

ICU, intensive care unit; SD, standard deviation.
