# Supplementary material for: Biofilm formation is a risk factor for mortality in patients with Candida albicans bloodstream infection—Scotland, 2012–2013
Source: Clin Microbiol Infect. 2016 Jan;22(1):87–93. doi: 10.1016/j.cmi.2015.09.018 (PMC4721535; doi:10.1016/j.cmi.2015.09.018)
Supplement: Supplementary file 1 [file mmc1.docx]

**Supplementary Figure 1.** **Correlation of three different biofilm quantification methods.** *Candida albicans* biofilm biomass (24h [n=107]) assessed by crystal violet staining was compared with XTT metabolic assay (blue circle) and SYTO 9 fluorescence measurement (green circle). The trend lines in the graph shows a positive correlation between both CV and XTT assay (blue line, Pearson correlation r= 0.778) and CV vs SYTO9 assay (green line, Pearson correlation r= 0.602).


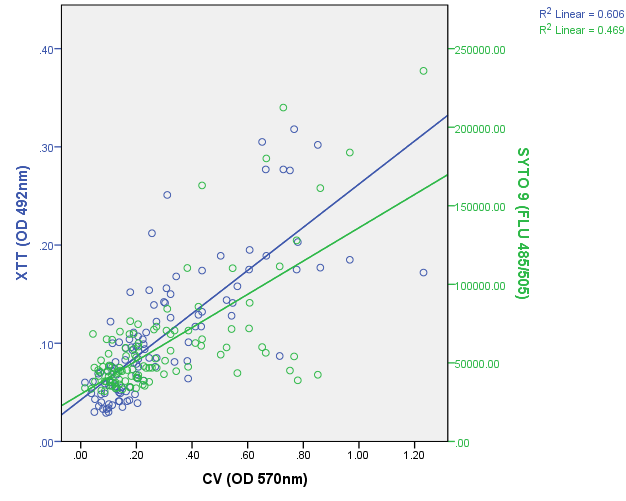


| Comparison | Analysis | Variables | Statistical significance (p) |
| --- | --- | --- | --- |
| *C. albicans* Vs *C. glabrata* | Kaplan-Meier  Log rank test |  | 0.623 |
|  | Univariable Cox regression analysis | Age | 0.260 |
|  | Univariable Cox regression analysis | Line removal | 0.174 |
|  | Multivariable Cox regression analysis | Age & Line removal | 0.051 |
| *C. albicans* HBF Vs LBF | Kaplan-Meier  Log rank test |  | 0.268 |
|  | Univariable Cox-regression analysis | Age | 0.132 |
|  | Univariable Cox-regression analysis | Parenteral nutrition | 0.211 |
|  | Multivariable Cox-regression analysis | Age and Parenteral nutrition | 0.026 |
| Comparison | Analysis | Variables | Statistical significance (p) |
| *C. albicans* Vs *C. glabrata* | Kaplan-Meier  Log rank test |  | 0.623 |
|  | Univariable Cox regression analysis | Age | 0.260 |
|  | Univariable Cox regression analysis | Line removal | 0.174 |
|  | Multivariable Cox regression analysis | Age & Line removal | 0.051 |
| *C. albicans* HBF Vs LBF | Kaplan-Meier  Log rank test |  | 0.268 |
|  | Univariable Cox-regression analysis | Age | 0.132 |
|  | Univariable Cox-regression analysis | Parenteral nutrition | 0.211 |
|  | Multivariable Cox-regression analysis | Age and Parenteral nutrition | 0.026 |

**Supplementary Table 1**
